# Synthesis and Characterization of Diketopyrrolopyrrole-Based Aggregation-Induced Emission Nanoparticles for Bioimaging

**DOI:** 10.3390/molecules27092984

**Published:** 2022-05-06

**Authors:** Geonho Lee, Jongwook Park, Seong Hyun Jang, Sang Yoon Lee, Jihyun Seong, Jae Woong Jung, Kyobum Kim, Tae Gyu Hwang, Jun Choi

**Affiliations:** 1 Material & Component Convergence R&D Department, Korea Institute of Industrial Technology (KITECH), Ansan 15588, Korea; geonho118@kitech.re.kr (G.L.); seonghyun@kitech.re.kr (S.H.J.); sangyoon@kitech.re.kr (S.Y.L.); 2 Department of Chemical Engineering, KyungHee University, Yongin 17104, Korea; jongpark@khu.ac.kr; 3 Laboratory of Organic Photo-Functional Materials, Department of Materials Science and Engineering, Seoul National University, 1 Gwanak-ro, Gwanak-gu, Seoul 08826, Korea; 4 Department of Advanced Materials Engineering for Information & Electronics, Integrated Education Institute for Frontier Science & Technology BK21 Four, KyungHee University, Yongin 17104, Korea; wodndwjd@khu.ac.kr; 5 Department of Chemical and Biochemical Engineering, Dongguk University, Seoul 04620, Korea; jh.seong520@gmail.com; 6 Research Center for Advanced Specialty Chemicals, Korea Research Institute of Chemical Technology (KRICT), Ulsan 44412, Korea

**Keywords:** dyes, aggregation-induced emission, nanoparticles, fluorescence, dispersibility, bioimaging

## Abstract

Conventional fluorescent dyes have the property of decreasing fluorescence due to aggregation-caused quenching effects at high concentrations, whereas aggregation-induced emission dyes have the property of increasing fluorescence as they aggregate with each other. In this study, diketopyrrolopyrrole-based long-wavelength aggregation-induced emission dyes were used to prepare biocompatible nanoparticles suitable for bioimaging. Aggregation-induced emission nanoparticles with the best morphology and photoluminescence intensity were obtained through a fast, simple preparation method using an ultrasonicator. The optimally prepared nanoparticles from 3,6-bis(4-((*E*)-4-(bis(40-(1,2,2-triphenylvinyl)-[1,10-biphenyl]-4-yl)amino)styryl)phenyl)-2,5-dihexyl-2,5-dihydropyrrolo[3,4-c]pyrrole-1,4-dione (DP-R2) with two functional groups having aggregation-induced emission properties and additional donating groups at the end of the triphenylamine groups were considered to have the greatest potential as a fluorescent probe for bioimaging. Furthermore, it was found that the tendency for aggregation-induced emission, which was apparent for the dye itself, became much more marked after the dyes were incorporated within nanoparticles. While the photoluminescence intensities of the dyes were observed to decrease rapidly over time, the prepared nanoparticles encapsulated within the biocompatible polymers maintained their initial optical properties very well. Lastly, when the cell viability test was conducted, excellent biocompatibility was demonstrated for each of the prepared nanoparticles.

## 1. Introduction

Fluorescent dyes are used for various applications in our everyday life, for example, in light-emitting diodes, solar cells, photodetectors, and bioimaging [1,2,3,4]. In particular, in the development of modern healthcare technologies, bioimaging, and biosensing are emerging as essential tools for monitoring molecular-level processes during research in medicine and biology and for disease diagnosis and treatment. Introducing fluorescent dyes for bioimaging offers advantages such as simple processing, high sensitivity, multiple parameter non-destructive detection, real-time monitoring, and quantification [5,6,7]. Most conventional fluorescent dyes have excellent quantum yields in a dilute solution. However, aggregation-caused quenching (ACQ) effects with increasing concentration constitute a major obstacle to the development of biodiagnostic kits or cell imaging techniques. ACQ can result in fluorescence intensities being drastically diminished because of π–π stacking interactions that occur when the dye molecules aggregate at high concentrations and in the solid state [8]. In 2001, Tang et al. were the first to report dye molecules that exhibited aggregation-induced emission (AIE), a property that results in an increase in the brightness of fluorescence as the fluorophore concentration increases or as they aggregate into solid-phase particles [9,10]. Unlike conventional ACQ dyes, for AIE dyes, molecules in a dilute solution have structures that include several aromatic groups that are freely rotating; thus, at high concentrations and in the solid state, non-radiative decay is blocked due to the restriction of intramolecular rotation (RIR) caused by π–π stacking interactions between molecules. Therefore, AIE dyes exhibit stronger fluorescence intensities when they are more aggregated, overcoming any ACQ effects [11].

The successful implementation of bioimaging requires fluorescent probes that have excellent fluorescence stability, high definition, water dispersibility, and biocompatibility [12,13,14,15]. Inorganic quantum dots (QDs) have excellent optical properties, and they can be rendered water-soluble and biocompatible by surface modification [16,17]. However, QDs can have toxic effects when heavy metals leak from them as a result of surface coating damage [18]. Gold nanoparticles (AuNPs) have excellent biocompatibility and fluorescence stability but weak photoluminescence (PL) intensity [19,20,21]. In particular, both QDs and AuNPs suffer from the above-mentioned ACQ problem. Therefore, AIE dyes are a good alternative as fluorescent labels for bioimaging as these organic phosphors have greater biocompatibility than QDs and exhibit an intense fluorescence emission even when aggregated.

In this study, the diketopyrrolopyrrole (DPP) compounds, DP-O, DP-R, and DP-R2 (see Section 2.1 for systematic names), were synthesized as AIE dyes by introducing triphenylamine (TPA) and tetraphenylethene (TPE). These rotational groups impart the capacity for AIE and emitting long-wavelength to DPP compounds, which itself exhibits durability and fluorescence. In addition, a fast, simple one-pot manufacturing method for obtaining nanoparticles (NPs) with uniform size and shape using an ultrasonicator was proposed. Because this approach involved long-wavelength AIE dyes, this method has various advantages, such as minimal optical damage to biological samples during bioimaging, minimal interference from scattering and tissue autofluorescence, and excellent tissue permeability [22,23,24]. The prepared AIE NPs were shown to have excellent biocompatibility and water dispersibility thanks to the fact that they were encapsulated by Pluronic F-127 and poly(methyl methacrylate) (PMMA). Moreover, the fluorescence stability of the dye molecules was remarkably enhanced because the encapsulating polymers protected the AIE dyes from being photolyzed by oxygen and other chemical species [25,26,27,28].

We were able to obtain AIE NPs with optimized PL intensity and particle shape by tuning the concentration of AIE dyes and the proportions of their various constituent components, such as the surfactant and capping agent; the optimal conditions were achieved by comparing the results of several different trial syntheses. The prepared NPs had a uniform particle size distribution, and it was found that, like the AIE dyes themselves, the NPs prepared from the AIE dyes had excellent AIE properties as well. Upon comparing the PL intensities in solution over time before and after NP encapsulation, it was observed that while the PL intensity of the AIE dyes decreased rapidly, the AIE NPs encapsulated by Pluronic F-127 and PMMA exhibited stable fluorescence, with their PL intensity being maintained at a constant level for >24 h. Their morphology, optical properties, and cell viability were investigated and compared thoroughly.

## 2. Materials and Methods

### 2.1. Materials

A series of diketopyrrolopyrrole (DPP) compounds, 3,6-bis(4′-(diphenylamino)-1,1′-biphenyl-4-yl)-2,5-dihexyl-2,5-dihydropyrrolo[3,4-c]pyrrole-1,4-dione (DP-O), (*E*)-3-(4-(4-(bis(40-(1,2,2-triphenylvinyl)-[1,10-biphenyl]-4-yl)amino)styryl)phenyl)-6-(4-bromophenyl)-2,5-dihexyl-2,5-dihydropyrrolo[3,4-c]pyrrole-1,4-dione (DP-R), and 3,6-bis(4-((*E*)-4-(bis(40-(1,2,2-triphenylvinyl)-[1,10-biphenyl]-4-yl)amino)styryl)phenyl)-2,5-dihexyl-2,5-dihydropyrrolo[3,4-c]pyrrole-1,4-dione (DP-R2), were synthesized via procedures reported in the literature [29,30,31,32]. Pluronic F-127, poly(methyl methacrylate) (PMMA, average M_W_ ~120,000), sodium dodecyl sulfate (SDS, ≥98.5%), ultrapure water (nuclease-free water), chloroform (anhydrous, ≥99%), tetrahydrofuran (anhydrous, ≥99.9%), and sodium hydroxide (ACS reagent, ≥97.0%) were purchased from Sigma-Aldrich (Burlington, MA, USA). All chemicals were used without further purification.

### 2.2. Preparation of AIE NPs

The AIE NPs were prepared using a modified emulsion–solvent evaporation method [33]. First, 0.8 mL of Pluronic F-127/PMMA and chloroform were combined, sealed, and completely dissolved by stirring at 45 °C. Then, after 0.5 mg of each of the synthesized AIE dyes (DP-O, DP-R, and DP-R2) was completely dissolved in 0.2 mL of chloroform, 0.8 mL of the prepared Pluronic F-127/PMMA stock solution was added, and a homogeneous mixture was obtained. Each mixture (500 μL) was added to the SDS solution (2.4 mL) and rapidly mixed using a vortex mixer. It was then emulsified using an ultrasonicator (SONOPULS series HD 2000.2, Bandelin Co.; at % amplitude, 3 min), and the chloroform was completely removed at 45 °C using a vacuum rotary evaporator. The mixture was centrifuged at 12,000 rpm for 20 min to obtain a precipitate. The obtained precipitate was washed twice using a NaOH solution (0.01 M) to inactivate bacteria/viruses and for purification. Lastly, purified AIE NPs were obtained by centrifugation after redispersion in ultrapure water, and they were stored at 4 °C in the dark. The different proportions of the various contents used to prepare the AIE NPs are listed in Table 1.

### 2.3. Characterization of AIE NP Size and Morphology

Dynamic light scattering (DLS; DLS 7000, Otsuka Electronics Co., Ltd., Osaka, Japan) analysis was used to obtain the size distributions of the AIE NPs. The morphology of the AIE NPs was analyzed using field-emission scanning electron microscopy (FE-SEM; SU8000, HITACHI, Tokyo, Japan) operating at 5 kV.

### 2.4. Optical Property Characterization

Photoluminescence (PL) spectra and fluorescence stability evaluations were performed using a fluorescence spectrometer (FS-2, Scinco, Seoul, Korea) and a quartz cuvette with a 10-mm path length. The excitation wavelength of the device was 365 nm. UV–Vis absorption spectra of the AIE NP aqueous solutions were obtained using a UV/Vis spectrometer (Mega-800, Scinco) and a quartz cuvette with a 10-mm path length. In this study, all the optical property data were acquired from samples with concentrations of 1 × 10^−5^ M for the AIE dyes or 0.5 mg/mL for Mega-800 he AIE NPs. To examine the fluorescence stability, the fluorescence intensity over time for the prepared AIE dyes and AIE NPs dispersed in THF and water, respectively, were compared.

### 2.5. Cell Viability

The cytotoxicities of the DP-O, DP-R, and DP-R2 AIE NPs were evaluated using a WST-1 assay. HeLa cells were cultured in high-glucose Dulbecco’s modified Eagle’s medium (DMEM, Hyclone Laboratories, Inc., Logan, UT, USA) with 10% fetal bovine serum (FBS, Hyclone) and 1% penicillin/streptomycin and then incubated (37 °C, 5% CO_2_, 90–95% relative humidity). Subsequently, the HeLa cells were seeded in a 96-well plate; 160 μL of a solution of 5 × 10^4^ cells/mL was used for each cell. After 24 h, the cells were incubated with different amounts of AIE NPs (DP-O, DP-R, or DP-R2) for 12 or 24 h. Then, the cells were washed with Dulbecco’s phosphate-buffered saline (DPBS) three times. Then, 10 μL of the WST-1 cell proliferation reagent (EZ-Cytox cell viability assay kit, DAEIL Lab, Korea) and 100 μL of DMEM cell culture medium were added to each well and the cells were then incubated for another 3 h at 37 °C in the dark. Finally, the optical density (OD) of each of the solutions was monitored at 450 nm using a microplate reader (iD3, SpectraMax, San Jose, CA, USA), and the percentage cell viability was calculated with reference to the measured OD of the control group. GraphPad Prism 7.0 was used to conduct the statistical analysis (GraphPad Software Inc., San Diego, CA, USA). In the case of quantitative data, experiments were carried out in triplicate (*n* = 3) and assessed using one-way analysis of variance (ANOVA) and Tukey’s multiple-comparison test. The mean standard deviation is shown in the graphs. Statistical difference was considered when the *p*-value was less than 0.05 (*p* < 0.05).

## 3. Results and Discussion

**Scheme 1:**
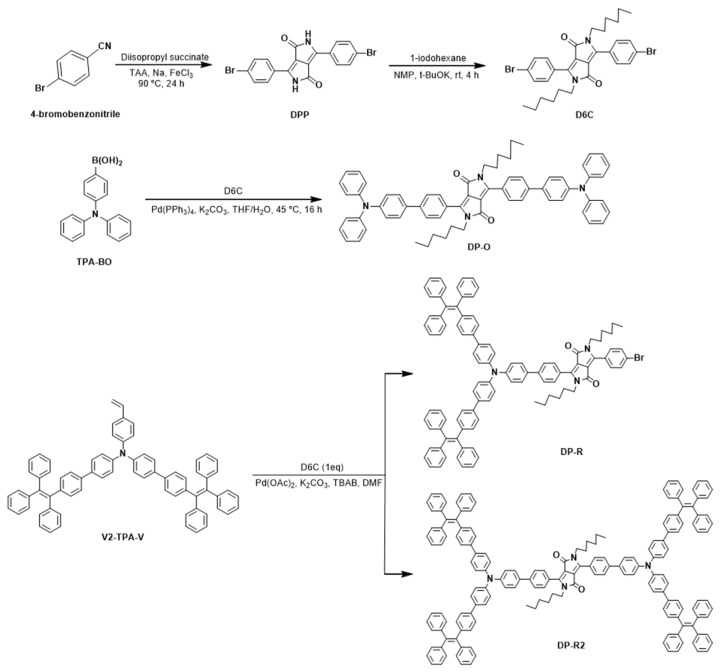
Synthetic route of DPP-based AIE dyes.

**Figure 1:**
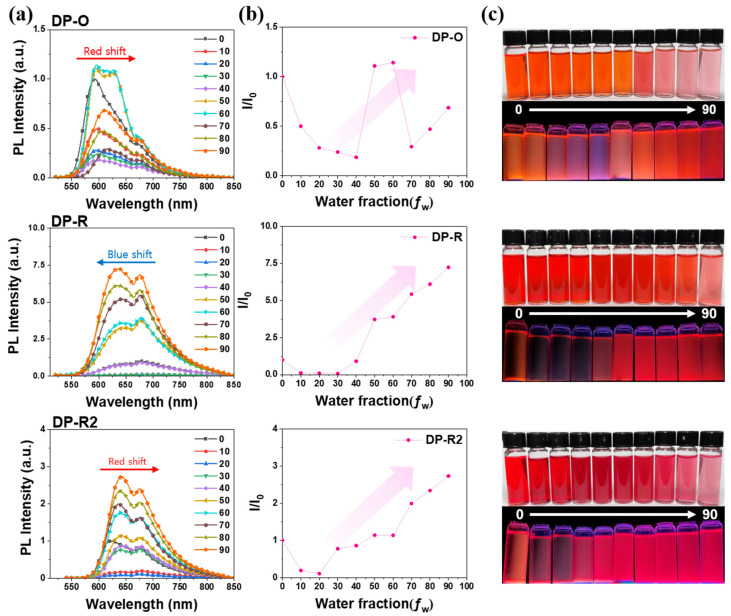
(**a**) PL spectra, (**b**) intensity change of maximum emission wavelength, and (**c**) photographs by water fraction (*f*_w_) of DPP-based AIE dyes. (Excitation wavelength = 365 nm).

The AIE dyes used in the synthesis of the AIE NPs were DP-O, DP-R, and DP-R2, and the synthetic routes for these dyes are shown in Scheme 1. In general, substances that exhibit AIE are weakly fluorescent due to intramolecular rotation-induced non-radiative decay in a good solvent, such as THF. However, as the volume of water, a poor solvent, increases, the emitting fluorescence intensity increases with particle aggregation. Because the rotational groups TPA and TPE were introduced to the prepared dyes in this study, upon aggregation, intramolecular rotation of the molecules was limited by the π–π stacking interactions between molecules. Thus, non-radiative decay is suppressed, and it has been reported that the fluorescence increases to the extent that almost all the energy absorbed by the molecules is emitted as PL [34]. To verify the AIE properties of the synthesized dyes, we observed variation in the luminous intensity as the ratio of the good solvent, THF, and a poor solvent, water was varied, with the dye concentration fixed at 1 × 10^−5^ M. Figure 1 shows PL spectra, relative PL intensity plots, and photographs of the DPP AIE dyes. The initial PL intensity I_0_ is the fluorescence when the water fraction *f*_w_ is 0%, and I is the PL intensity when *f*_w_ > 0%. For DP-O, the PL intensity rapidly decreased over the range of *f*_w_ = 0–40%, but it was dramatically increased at *f*_w_ = 50–60%; in the latter range, the PL intensity was up to 1.1 times that of the initial value. The PL intensity over the range of *f*_w_ = 70–90% was slightly reduced with respect to that at *f*_w_ = 50–60%, but the dyes still exhibited AIE. As for DP-R and DP-R2, in both cases, the PL intensity decreased as *f*_w_ increased in the range of 0–20% but steadily increased over the entire region in which *f*_w_ > 20% because of the AIE effect; the maximum PL intensities were 7.2 and 2.7 times the initial PL intensities, respectively. Thus, in general, as the poor solvent fraction *f*_w_ increased, the molecular rotation of molecules was limited by the aggregation of molecules and the PL intensity consequently increased, i.e., AIE occurred. For DP-O and DP-R2, a red shift of the fluorescence was observed as *f*_w_ increased, but for DP-R, a blue shift occurred. For fluorescent dyes in general, donor–acceptor twisting occurs in a polar solvent such as water. Thus, weak luminescence that has been red-shifted by the dominant non-radiative decay is observed as twisted intramolecular charge transfer (TICT) occurs with the dyes aggregated. However, in the cases of DP-O and DP-R2, which exhibit AIE, strong red-shifted luminescence by TICT and RIR was observed [35]. By comparison, for DP-R, PL intensity was found to increase with *f*_w_ due to the AIE effect, but conformational twisting between TPA moiety and DPP was suppressed due to the structurally ordered aggregation. Therefore, as the TICT state is reduced, the PL spectrum is deduced to have a blue shift. [36,37]. Consequently, we confirmed that these three types of prepared DPP dyes exhibited strong AIE, suggesting that they might be suitable but need to be comparatively investigated for incorporation in long-wavelength AIE NPs with excellent biocompatibility and water dispersibility.

**Figure 2:**
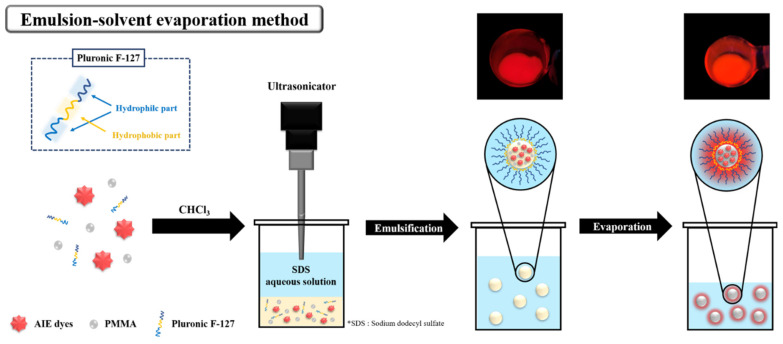
Schematic illustration of DPP-based AIE NPs prepared by emulsion solvent evaporation method.

**Figure 3:**
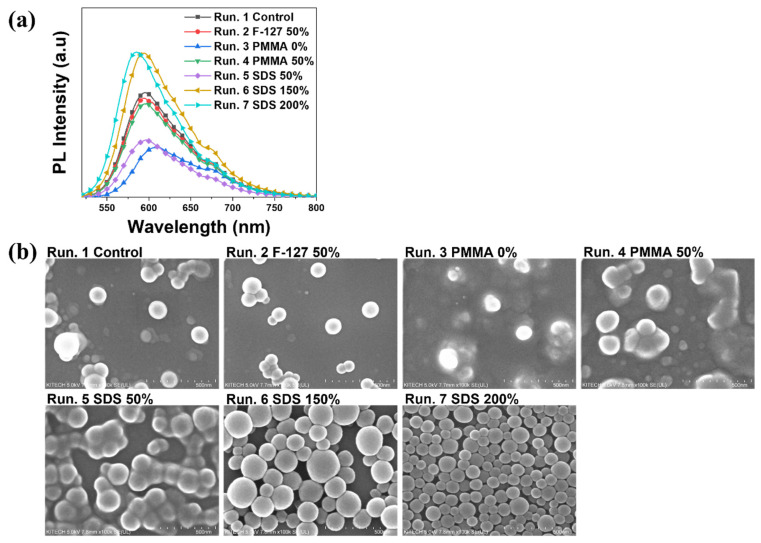
(**a**) PL spectra and (**b**) SEM images of DPP-based AIE NPs prepared by Table 1 recipes. (Concentration of 0.5 mg/mL DP-O).

In this study, AIE NPs were produced via the emulsion–solvent evaporation method using an ultrasonicator. A schematic illustration of this method is shown in Figure 2. Pluronic F-127, a biocompatible polymer, is an amphiphilic triblock copolymer that includes hydrophilic and hydrophobic moieties. The hydrophobic part of the polymer wrapped around the AIE dyes to form micelles with hydrophilic exteriors. First, to optimize the PL intensity and shapes of the AIE NPs, PL spectra and SEM images of AIE NPs prepared with different content ratios (Table 1) were compared, using DP-O as the representative fluorescent dye (Figure 3). Pluronic F-127 was essentially used because it allows AIE NPs with high water dispersibility. The appropriate particle shapes could not be obtained without using the surfactant SDS. The Run 2 sample, with a 50% reduction in Pluronic F-127, and the Run 4 sample, with a 50% reduction in PMMA, did not exhibit significantly different PL intensities and particle shapes compared to a control, Run 1 sample. The Run 3 sample, for which PMMA was not used, showed the greatest reduction in PL intensity because, although some particles were formed, sufficient particle formation was not induced due to the lack of the PMMA for binding. In the case of the Run 5 sample, for which the SDS content was reduced by 50% with respect to the Run 1 sample, many particles were aggregated, and the PL intensity was decreased with respect to that of the Run 1 sample. It is thought that the average size of particles increased with aggregation because of the reduced amount of surfactant, and hence the ACQ effect dominated [33]. By comparison, for the Run 6 sample, for which 150% of the SDS content of the Run 1 sample was used, the particles showed very distinct shapes because a sufficient amount of surfactant was used; further, these particles were monodisperse and had the highest PL intensity. The PL intensity of the Run 7 sample, with 200% of the SDS content of the Run 1 sample, was not significantly different from that of the Run 6 sample, but a decrease in the average particle size was confirmed from the SEM image, and hence blue shift was observed. Thus, in descending order, the PL intensities were as follows: Run 6 ≈ Run 7 > Run 1 > Run 2 > Run 5 > Run 4 > Run 3. Because fluorescent probes with longer wavelengths are advantageous in terms of bioimaging utilization, we adopted the recipe used for Run 6, which generated NPs that emit light at a longer wavelength than those generated by Run 7 in our optimized method for preparing AIE NPs.

**Figure 4:**
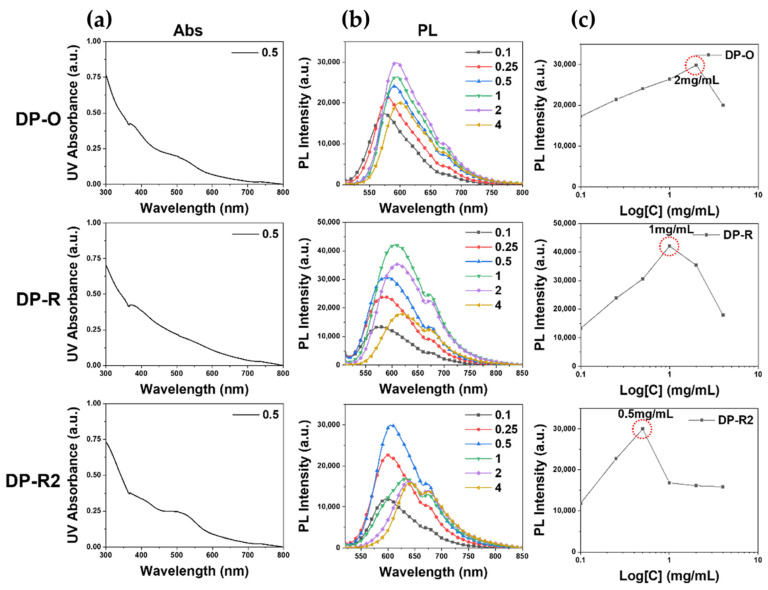
(**a**) UV/Vis spectra of DPP-based AIE NPs prepared at a concentration of 0.5 mg/mL, (**b**) PL spectra, and (**c**) intensity of maximum emission wavelength of AIE NPs by dye concentration. (Excitation wavelength = 365 nm).

**Figure 5:**
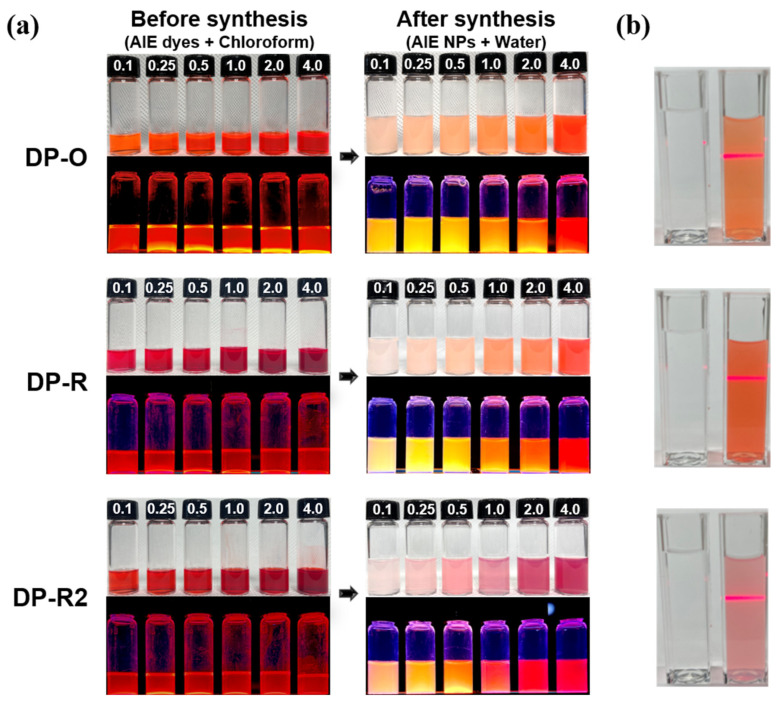
(**a**) Photographs before and after fabrication of DPP-based AIE NPs. (**b**) Tyndall phenomenon by AIE NPs under laser irradiation, the left cuvette is pure water, and the right cuvette is AIE NPs solution.

The UV–Vis absorption spectra of the DP-O, DP-R, and DP-R2 AIE NPs and the PL spectra obtained using different AIE dye concentrations are shown in Figure 4. All the UV–Vis absorption spectra of these AIE NPs, which were acquired using a concentration of 0.5 mg/mL, showed gradually increasing intensity as the wavelength shortened (Figure 4a). To obtain the optimal dye concentration, i.e., the highest PL intensity, for the preparation of the AIE NPs, the PL intensities of the AIE NPs with different DP-O, DP-R, and DP-R2 AIE dye concentrations were compared (Figure 4b,c). The amounts of the respective dyes used were 0.1, 0.25, 0.5, 1, 2, and 4 mg in the preparation of the different test samples of AIE NPs. The measurement results in the case of each of the three dyes showed that the higher the dye concentration, the more red-shifted the emission wavelength. The optimized dye concentrations were found to be 2 mg/mL for DP-O, 1 mg/mL for DP-R, and 0.5 mg/mL for DP-R2, respectively. It was considered that greater red shifting was observed as the concentration increased for all the three dyes because more molecules were involved in π–π stacking interactions, and this promoted intermolecular charge transfer. In addition, the long-wavelength shoulder of the emission band gradually increased as excimer formation was promoted with the increase in dye concentration. The photographs of the AIE dyes (before NP synthesis) and of the AIE NPs (after NP synthesis) in Figure 5a also demonstrate that the magnitude of the red shift increased with the dye concentration. Furthermore, it is apparent that although the AIE dyes dissolved in chloroform were weakly fluorescent, the fluorescence was rendered much more intense, thanks to the RIR mechanism, after the incorporation of the dye within the NPs. To verify the presence of small-sized particles in the AIE NP solutions, a laser was irradiated from the left after placing pure water in a cuvette on the left of another cuvette containing the prepared AIE NP solution, and the Tyndall effect was observed (Figure 5b). The Tyndall effect is a phenomenon in which light is scattered by colloids with small particle sizes. Thus, the formation of AIE NPs was verified indirectly by observing the Tyndall effect upon transmitting a laser through solutions containing the prepared AIE NPs [38].

**Figure 6:**
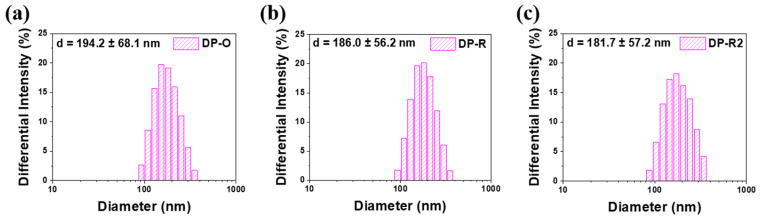
Size distribution of DPP-based AIE NPs using DLS. (**a**) DP-O, (**b**) DP-R, (**c**) DP-R2.

DLS analysis was performed to directly determine the particle size distributions of the three different AIE NPs prepared using the optimized synthesis method and dye concentrations, and the results are shown in Figure 6. All the samples had similar particle sizes and uniform size distributions: the measured average particle sizes were 194.2 ± 68.1 nm for DP-O, 186.0 ± 56.2 nm for DP-R, and 181.7 ± 57.2 nm for DP-R2, respectively.

**Figure 7:**
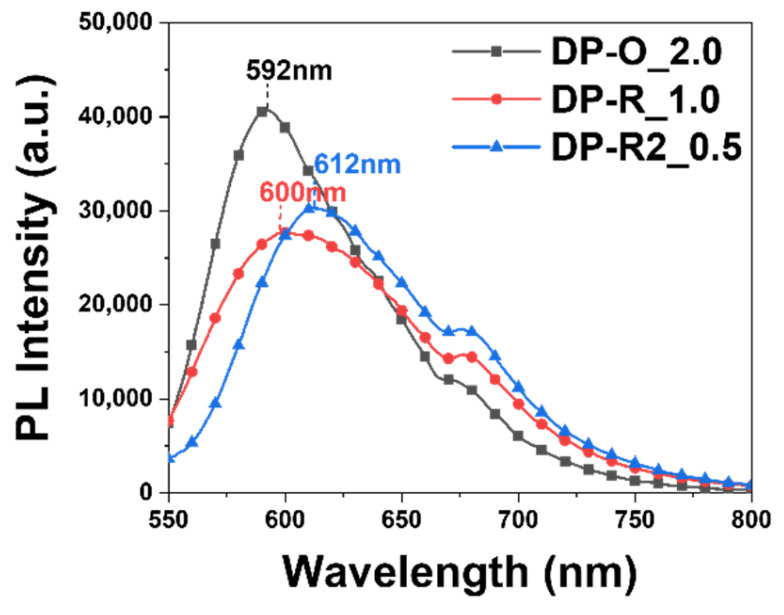
Comparison of PL spectra of DPP-based AIE NPs under the same measurement conditions.

Their PL spectra were compared again under the same conditions, and the optimized DP-O (2 mg/mL) AIE NPs were found to possess a greater PL intensity than the DP-R (1 mg/mL) and DP-R2 (0.5 mg/mL) AIE NPs, as shown in Figure 7. Comparisons of the PL intensities revealed that the PL intensities of DP-O and DP-R2, which have two AIE functional groups, were stronger than that of DP-R, which has one functional group with AIE properties. Thus, in descending order, the PL intensities were DP-O > DP-R2 > DP-R. The emission wavelength differed among DPP compounds as it was affected by the electron-donating capacity of the additional substituent and its position on the molecule. In previous studies, HOMO, LUMO, and electron density distributions of DPP compounds were analyzed through TD-DFT calculations [29,32]. Since the calculated band gap (ΔE) of the DPP compounds decreases in the order of DT-O (2.25 eV) < DT-R (2.09 eV) < DT-R2 (2.01 eV), it was confirmed that the maximum absorption wavelength (λ) was bathochromic shifted. In fact, it was confirmed that the maximum emission wavelength was 593 nm for DP-O AIE NPs, 600 nm for DP-R AIE NPs, and 612 nm for DP-R2 AIE NPs. This was because the electron-donating capacity of the substituent with TPE groups added to the end of the TPA groups introduced into DP-R and DP-R2 is stronger than that of DP-O with only TPA groups introduced. As a result, the PL spectra of DP-R and DP-R2 were shifted to a longer wavelength because the excited state became stable with respect to that of the ground state due to internal charge transfer (ICT). Therefore, the longer the wavelength, the better the tissue permeability; thus, it can be considered that DP-R2 has a relative advantage as a fluorescent probe for bioimaging.

**Figure 8:**
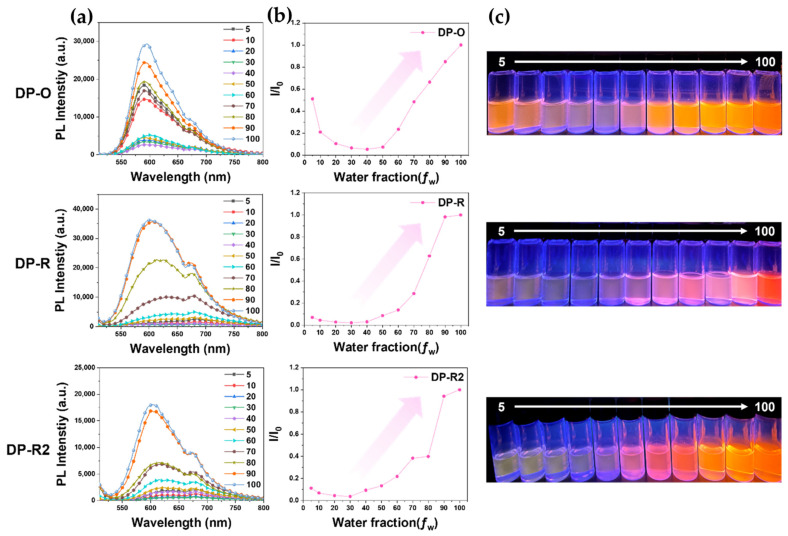
(**a**) PL spectra, (**b**) intensity change of maximum emission wavelength, and (**c**) photographs by water fraction (*f*_w_) of DPP-based AIE NPs. (Excitation wavelength = 365 nm).

To verify the AIE properties after nanoparticle formation, PL spectra for a range of different THF/water volume ratios were analyzed using an AIE NP at the concentration of 1 × 10^−5^ M, and the results are shown in Figure 8. Comparing this data with that in Figure 1, it is noteworthy that the AIE properties were even further reinforced after appropriate nanoparticle formation. Similar to the trend of the PL intensity of the AIE dyes versus the water fraction (*f*_w_), the PL intensities of all AIE NPs were the strongest at *f*_w_ = 100%, and they gradually decreased as *f*_w_ decreased. It seems that the polymers and AIE dyes comprising the AIE NPs were dissociated because of the increased THF content. Therefore, it is thought that the rate of non-radiative decay was increased by the aforementioned intramolecular rotation in the dyes, and the PL intensity decreased accordingly.

**Figure 9:**
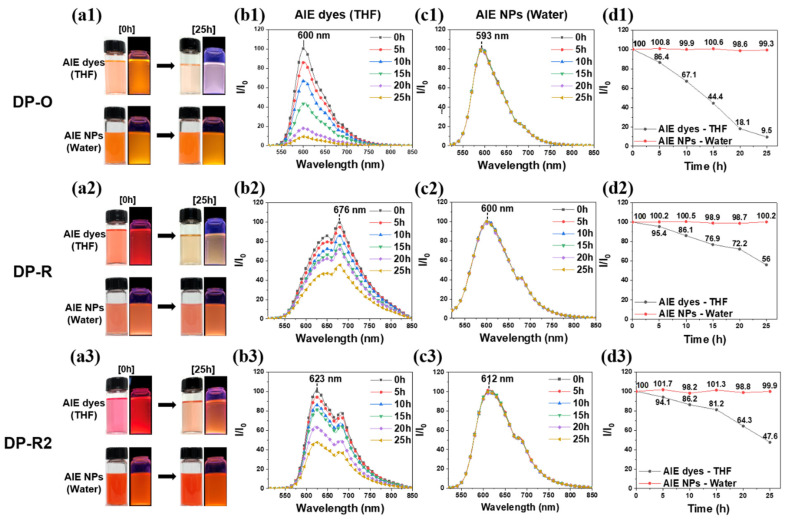
Fluorescence stability of DPP-based AIE dyes and AIE NPs solutions. (**a1**–**a3**) photographs of fluorescence intensity at 0 and 25 h, (**b1**–**b3**) PL spectra of AIE dyes over time, (**c1**–**c3**) PL spectra of AIE NPs over time, and (**d1**–**d3**) Comparison of PL intensity of AIE dyes and AIE NPs. (Excitation wavelength = 365 nm).

To examine the fluorescence stability of AIE NPs, the fluorescence intensity over time for the AIE dyes and AIE NPs dispersed in THF and water, respectively, were compared, as shown in Figure 9. In the case of AIE dyes in THF, after 25 h, the PL intensity had severely decreased to 9.5% for DT-O, 56.0% for DT-R, and 47.6% for DT-R2, respectively, compared to their initial PL intensity (100%). This is because the fluorescence properties of AIE dyes with a donor–acceptor (D–A) structure in solution are generally strongly affected by solvent polarity. Molecular polarization occurs when a D–A structure is excited, and solvent relaxation takes place: the surrounding solvent molecules are reorganized in accordance with the polarization of the dye molecules. At this point, in THF, a solvent with high polarity, charge transfer from donor to acceptor can occur easily, and the dye molecule system is stabilized, which consumes energy and lowers the S1 state. Consequently, light is emitted at a longer wavelength due to increased solvent relaxation in a polar solvent, and a PL intensity that rapidly decreases as the rate of non-radiative decay increases is observed. In contrast, the AIE NPs that were dispersed in water maintained their initial strong PL intensity, and very little intensity reduction was observed even after 25 h. As the AIE NPs were produced by encapsulating the AIE dyes in polymers, they were rendered water dispersible, which makes them suitable for bioimaging and also has the effect of limiting intramolecular rotation. Furthermore, the fluorescence stability of the prepared AIE NPs became remarkably high since they were protected by the biocompatible polymer passivation layer from external oxygen and other chemical species.

**Figure 10:**
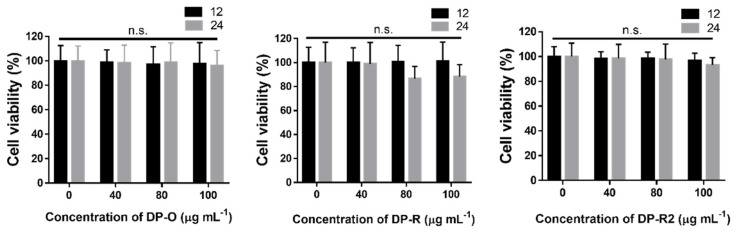
Biocompatibility estimation of AIE NPs at different concentrations with HeLa cells for 12 h and 24 h (*p* > 0.05, n.s.: no significance).

Lastly, a cell viability test was conducted to evaluate the potential of each fabricated AIE NP for use as a fluorescent probe for bioimaging, and the results of the WST-1 assay evaluation are shown in Figure 10. HeLa cells were seeded and incubated for 12 h and 24 h after adding AIE NPs at concentrations of 0, 40, 80, and 100 μg/mL to the cells, respectively. Subsequently, they were dyed and incubated at 37 °C for a further 3 h. As a result, the three types of AIE NPs were considered to be biocompatible, and statistically significant differences between the three samples were not observed in this assay. This is because the Pluronic F-127 used for encapsulation is highly biocompatible and the synthesized AIE organic dyes do not contain any harmful moieties.

## 4. Conclusions

In this study, three types of DPP-based fluorescent dyes having AIE properties were synthesized, and the possibility of using them for bioimaging was investigated. AIE NPs with water dispersibility and biocompatibility were prepared by encapsulating the synthesized fluorescent dyes in Pluronic F-127 and PMMA. The designed AIE NPs in this study emitted long-wavelength (>600 nm) light and were simply and rapidly prepared using an ultrasonicator in one pot via the emulsion–solvent evaporation method. The conditions corresponding to the highest PL intensity and best particle shape of the AIE NPs were determined by adjusting the component content of these biocompatible fluorescent probes. Monodisperse AIE NPs, with uniform particle sizes of 194.2 ± 68.1 nm for DP-O, 186.0 ± 56.2 nm for DP-R, and 181.7 ± 57.2 nm for DP-R2, were verified via SEM and DLS analyses. Observation of the measured PL spectra allowed us to establish that the PL spectrum variation was dependent on the chemical structure of the introduced fluorescent dye. In particular, the prepared AIE NP from DP-R2 with two functional groups having AIE properties and additional donating groups at the end of the TPA groups was considered to have the greatest potential as a fluorescent probe for bioimaging because the wavelength corresponding to the maximum of its emission spectrum was longest (612 nm), and it was also demonstrated to have a high PL intensity. Furthermore, it was found that the tendency for AIE, which was apparent in the dye itself, became much more marked after the dyes were incorporated within NPs. While the PL intensities of AIE dyes were observed to decrease rapidly over time, the AIE NPs encapsulated within the biocompatible polymers maintained their initial optical properties very well. Lastly, when the cell viability test was conducted, excellent biocompatibility was demonstrated for each of the prepared AIE NPs. Therefore, it is expected that in future studies the AIE NPs that were optimized for manufacture in this study will be proven to have excellent potential as a bioimaging fluorescent probe since they have intense and stable long-wavelength fluorescence, high water dispersibility, and biocompatibility.

## Figures and Tables

**Table 1:** Quantities of reagents used to prepare AIE NPs.

Sample	AIE Dye (DP-O) (mg/mL)	Pluronic F-127 (mg)	PMMA (mg)	SDS Solution (%, *w*/*w*)
Run 1	0.5	25.0	50.0	0.250
Run 2		12.5	50.0	0.250
Run 3		25.0	0	0.250
Run 4		25.0	25.0	0.250
Run 5		25.0	50.0	0.125
Run 6		25.0	50.0	0.375
Run 7		25.0	50.0	0.500

## Data Availability

Not applicable.

## Sample Availability

Samples of the compounds are not available from the authors.
